# Evaluation of antibacterial and anthelmintic activities with total phenolic contents of Piper betel leaves 

**Published:** 2014

**Authors:** Kazi Nahid Akter, Palash Karmakar, Abhijit Das, Shamima Nasrin Anonna, Sharmin Akter Shoma, Mohammad Mafruhi Sattar

**Affiliations:** 1 *Department of Pharmacy, Noakhali Science and Technology University, Sonapur, Noakhali-3814, Bangladesh*; 2 *Department of Pharmacy, Jahangirnagar University, Savar, Dhaka-1342, Bangladesh*

**Keywords:** *Anthelminticactivity*, *Antibacterialactivity*, *Leafextract*, *Piperbetel*, *Totalphenoliccontents*

## Abstract

**Objective:** The study was conducted to investigate the antibacterial and anthelmintic activities and to determine total phenolic contents of methanolic extract of *Piper betel *leaves.

**Materials and Methods: **The extract was subjected to assay for antibacterial activity using both gram positive and gram negative bacterial strains through disc diffusion method; anthelmintic activity with the determination of paralysis and death time using earthworm (*Pheritima posthuma*) at five different concentrations and the determination of total phenolic contents using the Folin-ciocalteau method.

**Results: **The extract showed significant (p<0.01) zone of inhibitions against gram positive *Staphylococcus aureus* [(6.77±0.25) mm] and Gram negative *Escherichia coli* [(8.53±0.25) mm], *Salmonella typhi* [(5.20±0.26) mm], *Shigella dysenteriae* [(11.20±0.26) mm] compared to positive control Azithromycin (ranging from 20.10±0.17 to 25.20±0.35 mm) while no zone inhibitory activity was found for both the extract and the standard drug against Gram positive *Bacillus cereus*. The extract also showed potent anthelmintic activity requiring less time for paralysis and death compared to the standard drug albendazole (10 mg/ml). At concentrations 10, 20, 40, 60 and 80 mg/ml, leaves extract showed paralysis at mean time of 9.83±0.60, 8.50±0.29, 6.60±0.17, 6.20±0.44 and 4.16±0.60; death at 11.33±0.88, 9.67±0.33, 7.83±0.17, 7.16±0.60 and 5.16±0.72 minutes, respectively. Whereas the standard drug showed paralysis and death at 19.33±0.71 and 51.00±0.23 minutes respectively. The extract confirmed the higher concentration of phenolic contents (124.42±0.14 mg of GAE /g of extract) when screened for total phenolic compounds.

**Conclusion: **As results confirmed potential antibacterial and anthelmintic activities of *Piper betel* leaves extract, therefore it may be processed for further drug research.

## Introduction

The exploration of new medicinal properties of various plant species has induced the attention of the scientists towards the biologically active compounds since the last couple of decades. The reason behind this is that the bioactive compounds possess potent pharmacological activities and have low or no toxicity (Rahman and Islam, 2013 ▶; Prashant et al., 2008 ▶). This emerged interest to plant-derived medicines is mainly due to the resistance caused by indiscriminate use of synthetic medicines as well as the on-going perception that green medicines are safer than the synthetic drugs having severe adverse effects (Jigna et al., 2006 ▶).

Although there are many antibacterial agents available in the field of medicine, in recent years multidrug resistance has been developed in human pathogenic microorganisms due to indiscriminate prescription and malpractice of commercially available antibiotics (Walsh, 2000 ▶, Obeidat et al., 2012 ▶). In the present perspective of the developing countries, synthetic drugs are not only expensive and incapable for curing diseases but also have fatal adverse effects (Opara et al., 2012 ▶). Thus, there is an urgent need to explore new antibacterial components with diverse chemical structures and novel mode of actions because of the increase in the incidence of new and re-emerging pathogenic diseases (Nair and Sumitra, 2008 ▶) to replace those which have lost their effectiveness. Furthermore, we know our traditional and folkloric medicine has been using higher and aromatic plants for the purpose of extending the shelf life of foods showing inhibition against bacteria and yeasts (Hulin, 1998 ▶). 

Several studies have reported that many herbs possess varying degree of antimicrobial activities (Kaufman et al., 1989 ▶; Kumar et al., 2007 ▶). Therefore, the natural medicinal plants may be a potent source of new antibacterial agents.

Helminthiasis is a disease in which a part of the body is infested with parasitic worms like Roundworms (Nematodes), Tapeworms (Cestodes) or Flukes (Trematodes) (Rafi et al., 2011 ▶). Although the worms reside in the gastrointestinal tract, sometimes may burrow into the liver and other organs (Adate et al., 2012 ▶). Drugs that either kill or expel infesting helminthes (worms) are known as anthelmintic. Since ancient times the medicinal properties of plants have been investigated for scientific advancement throughout the world due to their potent anthelmintic activities (Mali and Mehta, 2008 ▶; Akhtar et al., 2000 ▶). Some broad spectrum anthelmintics (e.g. Piperazine citrate, Albendazole) are effective against parasitic flat worms as well as nematodes. However, majority of drugs are limited in their action (e.g. Praziquantel) as resistance may be developed very quickly (Iqbal et al., 2001 ▶; Jackson and Coop, 2000 ▶) and also the toxicity problems may be occurred (Akhtar et al., 2000). Therefore, it is necessary to find out new medicinal plants having broad spectrum anthelmintic activity with less toxicity (Jabbar et al., 2007 ▶; Eguale et al., 2011 ▶).

Auto-oxidation of lipids and reactive nitrogen species (RNS) are the main sources of reactive oxygen species (ROS) in the forms of superoxide anions, hydroxyl radicals and hydrogen peroxide (Aruoma, 1996 ▶). Generation of these additional ROS and RNS by Ultraviolet (UV) radiation, smoking and drug metabolisms are likely to damage several cellular components such as lipids, proteins, nucleic acids, and DNAs through the oxidation or nitration processes (Sawa et al., 2000 ▶). On the other hand, these reactive oxygen species cause inflammation or lesion on different organs and are related with various degenerative diseases including cancer, ageing, arteriosclerosis, and rheumatism (Hasanuzzaman et al., 2013 ▶). Phenolic compounds from natural sources are well known for their metal chelating, reducing capability, hydrogen donating and most exceptionally capturing free radicals and stopping chain reactions (Hurrell, 2003 ▶). It was reported that the hydroxyl groups present in the phenolic compounds may directly contribute to the antioxidant activity and have a critical role in scavenging free radicals (Elmastas et al., 2007 ▶). As a result plants containing high level of polyphenols have attracted greater importance as natural antioxidants worldwide.

The *P. betel *Linn. (Betel leaf), commonly known as paan in Bangladesh is a vine belonging to the Piperaceae family and is largely distributed in tropical and subtropical regions of the world (Adate et al., 2012 ▶). Leaves of *P. betel *possess different types of activities such as antidiabetic, antiulcer, antiplatelet aggregation, antifertility, cardiotonic, antitumour, antimutagenic, respiratory depressant (Santhakumari et al., 2003 ▶; Lei, 2003 ▶; Majumdar et al., 2002 ▶; Adhikari et al., 1998 ▶). It is also used as carminative, stomachic, anthelmintic, tonic, and aphrodisiac (Adate et al., 2012 ▶). Several reports have revealed that the leaf of this plant possesses many beneficial bioactivities and its extract has a great potential to be used in developing commercial products (Balaji et al., 2011 ▶). This became the basis of selection of this plant and particularly the leaves. Thus, the evaluating study aims to assess the antibacterial activity, anthelmintic activity and also to determine the total phenolic components of *P. betel* leaves in an *in vitro *study model which may be helpful in developing new novel drugs. 

## Materials and Methods

**Collection and p** **reparation of plant material**

The leaves of *P. betel* were collected from Chittagong, Bangladesh in July 2012. The plant was identified by the taxonomist of Bangladesh National Herbarium, Mirpur, Dhaka, Bangladesh and a voucher specimen was deposited in the herbarium unit (Accession number: DACB 38091). The sun dried powdered leaves (500 mg) of *P. betel* was macerated in 2.5 L of 99.8% methanol (Merck KGaA, Darmstadt, Germany). After 15 days the solution was filtered using filter cloth and Whatman^®^ filter paper No. 1. The resulting filtrates were then evaporated in water bath maintained at 45°C to dryness and thus a blackish-green semisolid mass of the extract was obtained (yield 25 g).

**Reagents and chemicals**

All the solutions, reagents used in this study were of analytical grade. They were procured from Sigma Chemical Co. Ltd, (St. Louis, MO, USA) and E. Merck (Germany). 

**Evaluation of the antibacterial activity**

*Collection of microorganisms*

Five pathogenic bacterial strains were used as the test organisms for antibacterial screening of the plant extract. Among them *Staphylococcus aureus* & *Bacillus cereus* were gram positive and *Escherichia coli*, *Salmonella typhi* & *Shigella dysenteriae* were gram negative. All the stock cultures were collected from Poultry Research and Training Center, Chittagong Veterinary and Animal Science University, Chittagong-4202, Bangladesh.

*Preparation of media and maintenance of bacteria*

All the bacterial strains were grown and maintained on Muller Hilton agar (Hi media, India) media at 37°C and pH (7.3±0.2). The bacteria were sub-cultured overnight using Muller Hilton broth medium (Rahman and Islam, 2013 ▶).

*Antibacterial assay*

The antibacterial activity of methanolic extract of *Piper betel* leaves was determined by disc diffusion technique (National Committee for Clinical Laboratory Standards, NCCLS, 2002). The test organisms were transferred to the test tubes containing about 5 ml of melted and sterilized agar medium with the help of a sterilized transfer loop in an aseptic area. The test tubes were shaken by rotation to get a uniform suspension of the organisms. The bacterial suspension was immediately transferred to the sterilized Petri dishes. The Petri dishes were rotated several times clockwise and anticlockwise to assure homogenous distribution of the test organisms in the media. 

Sterilized Whatman^®^ paper discs (6 mm in diameter) were treated with desired concentration (400 µg/disc) of previously prepared methanolic solution of the extract using a micropipette and dried in air under aseptic condition and placed at the equal distance in a circle on the plate. These plates were kept at low temperature for 4-6 h and by this time the test materials diffuse from disc to surrounding medium. The same process was conducted for the negative control methanol and the positive control Azithromycin (30 µg/disc). All the experiments were conducted in triplicates. Then the plates were incubated for 24 h at 37°C. At the end of the period, the inhibition zone against each microorganism by plant extract was measured and analyzed by using one way ANOVA followed by paired *t*-test in SPSS version 18.0.

**Evaluation of the anthelmintic activity**

**Collection of worms**

The earthworms belonging to species *Pheritima posthuma *(Annelida), about 3-5 cm in length and 0.1- 0.2 cm in width weighing about 0.8-3.04 g, were collected from the moist soil of Noakhali Science and Technology University, Sonapur, Noakhali-3814, Bangladesh. 

*Reference drug*

For the evaluation of anthelmintic activity of *Piper betel*, the methanolic extract of leaves of the plant was tested in various doses in each group. Distilled water was used as control. Albendazole (Batch no: ALF0171, Square Pharmaceuticals Ltd., Bangladesh) was used as the standard drug for comparative study with methanolic extract.

*Anthelmintic assay*

The anthelmintic assay was carried out as per the method of Adate et al. (2012) ▶ with minor modifications. Here the anthelmintic activity was assessed using earthworms because of their anatomical and physiological resemblance with that of the intestinal roundworm parasites of human being (Chatterjee, 1967 ▶**, **Kumar et al., 2010 ▶). They are widely used as effective tools for anthelmintic study because of their easy availability (Sangeetha et al., 2010 ▶). Normal saline water was used to wash all the worms and to remove all fecal matters. Extracts were weighed and dissolved in 10 ml of distilled water to obtain the concentrations of 10, 20, 40, 60 and 80 mg/ml. Earthworms were divided into seven groups (each containing five worms) in petridish. The extract was applied to the petridishes and the time of paralysis and death was determined. When no movement of any sort could be observed except when the worms were shaken vigorously, was considered as paralysis time. Time for death of worms was recorded after ascertaining that worms neither moved when shaken vigorously nor when dipped in warm water (50°C) followed by fading away of their body colors.

**Determination of total phenolic content**

Total phenolic contents were determined by the Folin-ciocalteau method (Chanda et al., 2013 ▶) using Gallic acid as standard. The extract samples (0.5 ml of different dilutions) were mixed with Folin-ciocalteu reagent (2.5 ml, 1:10 diluted with distilled water) for 5 min and aqueous Na_2_CO_3_ (2 ml, 7.5 % w/v) was then added. The mixture was incubated for 20 minutes at room temperature. After 20 minutes the absorbance was measured at 760 nm by UV-spectrophotometer. The total phenolic content of the samples were measured using the standard curve prepared from Gallic acid solution with different concentrations (6.25, 12.5, 25, 50, and 100 µg/ml). The phenolic contents of the sample were expressed as mg of GAE (Gallic acid equivalent) / g of the extracts.

**Statistical analysis**

All data are presented as mean±standard deviation (SD) and were analyzed by One-way analysis of variance (ANOVA) (SPSS for windows, version 18.0, IBM corporation, NY, USA). The values were considered significantly different at p<0.05.

## Results

**Antibacterial activity of** ***P. betel*** **extract**

The results of antibacterial activity of methanolic extract of *P. betel* leaves are explained in Table 1. In general, the mean zone of inhibition produced by the reference antibiotic azithromycin was between 20 to 25 mm and was larger than that produced by the extract which was between 5 to 11 mm. The extract showed highest zone of inhibition against the Gram positive *S*.* aureus* (6.77±0.25 mm) and Gram negative *S. dysenteriae* (11.20±0.26 mm). Gram negative strains were found more sensitive than Gram positive organisms to the extract on an average. However, it was also revealed by the study that both the extract and the standard drug (azithromycin) were resistant against the Gram positive strain *B*.* cereus* (Abo-State et al., 2012 ▶). As the zone of inhibition of *P. betel* was very low thus the MIC (minimum inhibitory concentration) was not determined (Raju et al., 2013 ▶).

**Anthelmintic activity of** ***P. betel*** **extract**

The crude methanolic extract of *P. betel* leaves was used to evaluate the anthelmintic activity and the activity of the methanolic extract was compared to that of standard drug albendazole (Table 2). 

As shown in the Table 2, crude methanolic extract of leaves of *P. betel *revealed significant anthelmintic activity at the concentration of 10, 20, 40, 60 and 80 mg/ml in a dose dependent manner. It was also seen that at the concentration of 80 mg/ml the extract demonstrated shortest time of paralysis and death time. At the concentration of 80 mg/mL, the methanolic extract caused paralysis of *Pheretima posthuma *at 4.16±0.60 min and death at 5.16±0.72 min, while Albendazole (positive control) caused paralysis and death at 19.33±0.71 min and 51.00±0.23 min, respectively at 10 mg/mL. From the study, it was also clear that the time for paralysis and death decreases as the increasing of concentrations of the extract (Table 2). Therefore, the results demonstrate that methanolic extract of *P. betel* leaves possess wormicidal activity and thus may be used as an anthelmintic. 

**Table 1:** *In-vitro* antibacterial activity of *P. betel* leaves

**Bacterial strain**	**Test organism**	**Zone of inhibition (mm)**	
		***P. betel*** ** (400 µg/disc)**	**Azithromycin (50 ** **µ** **g/disc)**
**Gram positive**	*Staphylococcus aureus*	6.77±0.25**	23.50±0.5
	*Bacillus cereus*	-	-
**Gram negative**	*Escherichia coli*	8.53±0.25***	25.20±0.35
	*Salmonella typhi*	5.20±0.26***	20.10±0.17
	*Shigella dysenteriae*	11.20±0.26**	24.97±0.15

Data are shown as mean ± standard deviation (SD) for triplicate of concentration. Different superscripts shown in the data indicate that the values are significantly different (paired t-test, p*<*0.05) from each other.

*** p<0.001,

** p<0.01 SPSS for windows, version 18.0).

**Determination of total phenolic contents of** ***P. betel*** **extract**

Table 3 shows the total phenolic contents of methanolic extracts of *P. betel* leaves. Total phenolic compounds were reported as gallic acid equivalents by reference to a standard curve (y=0.002x+0.107; R² = 0.889). The results showed that the total phenol contents of methanolic extract was found 124.42±0.14 mg of GAE/ g. of extract. The results of total phenolic contents suggest that the plant may possess good antioxidant activity.

**Table 2:** *In-vitro* Anthelmintic activity of *P. betel* leaves

**Group**	**Concentration** **(mg/ml)**	**Time taken for** **paralysis (min)**	**Time taken for** **death (min)**
**Control (Distilled water) **	-	-	-
**Standard (Albendazole)**	10	19.33±0.71	51.00±0.23
**Methanolic extract**	10	9.83±0.60**	11.33±0.88**
	20	8.50±0.29***	9.67±0.33***
	40	6.60±0.17***	7.83±0.17***
	60	6.20±0.44***	7.16±0.60***
	80	4.16±0.60***	5.16±0.72***

Values are expressed as mean ± standard deviation (SD). Values were found out by using ONE way ANOVA followed by Paired *t *-test. Significance level

** p˂0.01,

*** p˂0.001

**Table 3:** Determination of total phenolic contents of *P. betel *leaves

**Extract**	**Sl. No.**	**Absorbance of the sample**	**Average Absorbance**	**Total Phenolic Content (mg of** **GAE / gm.) of Extracts**
**Mthanolic Extract**		0.605	0.605±0.0006	124.42±0.14
		0.604		
		0.605		

Data represents mean ± standard deviation (n=3) of duplicate analysis.

## Discussion

Since decades, plants have proved to be a vital source of drug and many plants have been screened whether they contain compounds with therapeutic activity or not (Rosy et al., 2010 ▶). Therefore, it is very essential to evaluate the antibacterial activity of *P. betel*. The bacterial strains used in the current study were selected because of their clinical importance as they develop resistance against different antibiotics with their frequent uses. In the present study it was found that the mean zone of inhibition produced by the commercial antibiotic azithromycin was larger than that produced by methanolic extract of *P. betel* leaves. This fact may be explained by the postulate that the crude form of plant extract contains a lower concentration of bioactive compounds (Baravalia et al., 2009 ▶). While screening medicinal plants for antibacterial activity, it is generally expected that a greater number of compounds would be active against Gram positive rather than Gram negative bacteria (Joshi et al., 2011 ▶). However, in the present study it was found that the extract of *P. betel* was effective against both gram positive and gram negative bacteria which suggest that the plant extract may possess broad spectrum of antibiotic compounds or simply general metabolic toxin (Mohammed et al., 2010 ▶). In a research conducted by Balaji et al. (2011) ▶ using aqueous and ethanolic extract of leaves of *P. betel* indicated that the ethanolic extract of this plant showed better antibacterial activity against *B. subtilis, S. aureus* and *E. coli* and moderate activity against *M. luteus* and *P. aeruginosa*. These results are quite similar to that of our present study although the sample preparation and some organisms were different. This may be described by the fact that the secondary metabolites responsible for demonstrating antibacterial activity are greatly dependent on solvent system and collection process of metabolites from the plant sources (Rahman and Islam, 2013 ▶). Moreover, the geographical area and environment also affects the chemical composition of the plants and leads to the variation in activity (Girish and Satish, 2008 ▶). Again, it was reported by several studies that several phytochemicals like terpenoids, flavonoids, tannins, alkaloids, steroids and some phenolic compounds are responsible for the antibacterial activity of the plant extract (Ramzi et al., 2008 ▶; Sule et al., 2011 ▶). 

A study conducted by Al-Adhroey et al. (2011) ▶ showed that the methanolic extract of the *P. betel* leaves contains certain phytochemicals like alkaloids, terpenes, anthraquinones, flavonoids, tannins, saponins and steroids. Whatever the mechanism, it is clear that some of these phytoconstituents of the plant extract may be responsible for the antibacterial activity.

We know that parasitic helminthes affect human being and animals causing a chronic and debilitating disease which ultimately leads to death. Again, our traditional medicines hold a great promise as a great source of easily available effective anthelmintic agents to the people especially in developing countries. Many plants have reported to possess anthelmintic activity *in vitro *and *in vivo* (Kumar et al., 2010 ▶). In the current study observations were made for the time taken to paralysis and death of individual worms against the plant extract and the standard drug that is albendazole. It causes degenerative alterations in the tegument and intestinal cells of the worm by binding to the colchicine-sensitive site of tubulin, thus inhibiting its polymerization or assembly into microtubules. The loss of the cytoplasmic microtubules leads to impaired uptake of glucose by the larval and adult stages of the susceptible parasites and depletes their glycogen stores and ultimately leads to death (Mali and Wadekar, 2008 ▶; Mute et al., 2009 ▶). 

The present study also revealed that leaves of *P. betel *showed potent anthelmintic activity. This may be described by the fact that several compounds like alkaloid, polyphenol, flavonoid and terpene etc. may be responsible for the anthelmintic activity of the plant (Bate-Smith, 1962 ▶). Several studies have confirmed that the leaves of *P. betel* are abundant of various phytochemicals (Adate et al., 2012 ▶; Al-Adhroey et al., 2011 ▶) among which some may be responsible for the wormicidal activity. These compounds may act on the CNS of the parasites causing paralysis and death of worms or interfere with the energy generation in the helminthes by uncoupling the oxidative phosphorylation or they bind to free proteins in the gastrointestinal tract of the host animal or to glycoprotein on the cuticle of the parasite and causes death (Salhan et al., 2010 ▶).

The present study also estimated the phenolic contents of methanolic extract of *P. betel *leaves. It was reported that *P. betel *is a powerful source of both phenolic compounds and also of other phenolic acids such as gallic acid, gentisic acid, catechin and epicathecin (Sundang et al., 2012 ▶). Studies have also showed that the different levels of antioxidant activities in plants may be due to not only differences in their phenolic contents, but also in their phenolic acid components (Horax et al., 2005 ▶). Because of the hydroxyl groups in the phenolic compounds, they may directly contribute to the antioxidant activity and have a critical role in scavenging free radicals (Elmastas et al., 2007 ▶). Again, recent studies have shown that fruit and vegetable phenols and polyphenols such as flavonoids prevent free radical damage and lipid peroxidation (Bernardi et al., 2008 ▶). The high content of total phenolic components in the methanolic extract may have led to the better results found in the total antioxidant activity and free radical scavenging ability of the plant.

In conclusion from the recorded data, it is demonstrated that the methanolic extract of leaves of *P. betel* has promising antibacterial and anthelmintic effect. The extract also contains very prominent amount of phenolic compounds which may be responsible for its potent antioxidant activity. As the current study confirmed that leaves of *P. betel* showed several biological activities, so taking into consideration of all the findings it can be mentioned that *P. betel* leaves can contribute major role in drug research. The plant may be further explored for its phytochemical profile to recognize the active constituents accountable for its versatile activities. 
